# Genome-wide association study identified three major QTL for carcass weight including the *PLAG1-CHCHD7* QTN for stature in Japanese Black cattle

**DOI:** 10.1186/1471-2156-13-40

**Published:** 2012-05-20

**Authors:** Shota Nishimura, Kazunori Mizoshita, Ken Tatsuda, Tatsuo Fujita, Naoto Watanabe, Yoshikazu Sugimoto

**Affiliations:** 1 Shirakawa Institute of Animal Genetics, Japan Livestock Technology Association, Odakura, Nishigo, Fukushima, 961-8061, Japan; 2 Cattle Breeding Development Institute of Kagoshima Prefecture, Osumi, So, Kagoshima, 899-8212, Japan; 3 Hyogo Prefectural Institute of Agriculture, Forestry & Fisheries, Befu, Kasai, Hyogo, 679-0198, Japan; 4 Oita Prefectural Institute of Animal Industry, Kuju, Takeda, Oita, 878-0201, Japan; 5 National Livestock Breeding Center, Odakura, Nishigo, Fukushima, 961-8511, Japan

**Keywords:** GWAS, QTL, Carcass weight, Body size, Body weight, Growth trait, Cattle

## Abstract

**Background:**

Significant quantitative trait loci (QTL) for carcass weight were previously mapped on several chromosomes in Japanese Black half-sib families. Two QTL, *CW-1* and *CW*-*2*, were narrowed down to 1.1-Mb and 591-kb regions, respectively. Recent advances in genomic tools allowed us to perform a genome-wide association study (GWAS) in cattle to detect associations in a general population and estimate their effect size. Here, we performed a GWAS for carcass weight using 1156 Japanese Black steers.

**Results:**

Bonferroni-corrected genome-wide significant associations were detected in three chromosomal regions on bovine chromosomes (BTA) 6, 8, and 14. The associated single nucleotide polymorphisms (SNP) on BTA 6 were in linkage disequilibrium with the SNP encoding NCAPG Ile442Met, which was previously identified as a candidate quantitative trait nucleotide for *CW-2*. In contrast, the most highly associated SNP on BTA 14 was located 2.3-Mb centromeric from the previously identified *CW-1* region. Linkage disequilibrium mapping led to a revision of the *CW-1* region within a 0.9-Mb interval around the associated SNP, and targeted resequencing followed by association analysis highlighted the quantitative trait nucleotides for bovine stature in the *PLAG1-CHCHD7* intergenic region. The association on BTA 8 was accounted for by two SNP on the BovineSNP50 BeadChip and corresponded to *CW-3*, which was simultaneously detected by linkage analyses using half-sib families. The allele substitution effects of *CW-1*, *CW*-*2*, and *CW*-*3* were 28.4, 35.3, and 35.0 kg per allele, respectively.

**Conclusion:**

The GWAS revealed the genetic architecture underlying carcass weight variation in Japanese Black cattle in which three major QTL accounted for approximately one-third of the genetic variance.

## Background

Carcass weight is an economically important trait for livestock raised for meat production. Carcass weight is highly correlated with body weight and body size. We previously performed bovine quantitative trait locus (QTL) mapping using Japanese Black paternal half-sib families and detected significant linkages with carcass weight on bovine chromosome (BTA) 1, 6, 7, 10, and 14 [1]. Further analyses of the half-sib QTL revealed a carcass weight QTL on BTA 8. QTL on BTA 14, 6, and 8 were validated in another family and designated *CW-1CW2,* and *CW3*, respectively, of which *CW-1* and *CW2* were narrowed down to 1.1 Mb [2] and 591 kb [3], respectively.

The recent development of genomic tools such as the BovineSNP50 BeadChip [4] allowed us to perform a genome-wide association study (GWAS) in our bovine populations. This approach will improve the resolution of the QTL and enable us to estimate the allele frequency and effect size of a QTL in a given population. Several GWAS using the single nucleotide polymorphism (SNP) chips for bovine growth-related traits have been reported. Snelling et al. [5] detected the strongest association on BTA 6 and others on BTAs 7, 10, 11, 14, 20, and 23 that reached a Bonferroni-corrected significance level using more than 2500 crossbred beef cattle, although they did not indicate the portion of heritability that could be accounted for by the associated SNP. The association on BTA 6 corresponds to *CW-2*[3] and the orthologous region in humans includes the *NCAPG-LCORL* region that was identified as one of the loci associated with human adult height [6,7]. In bovine, *CW-2* was associated with body size; including body length, width, and height; and the association with withers height was strongest [8]. Pausch et al. [9] detected a Bonferroni-corrected significant association with body size only on BTA 14 using 1800 bulls of the German Fleckvieh breed. The association also corresponded to the loci for human adult height, including *PLAG1, MOS, CHCHD7, RDHE2, RPS20, LYN, TGS1,* and *PENK*[7,10]. They identified a highly significantly associated SNP in a putative polyadenylation signal of *RPS20*. Karim et al. [11], however, recently identified causative variations influencing bovine stature in the *PLAG1-CHCHD7* intergenic region. This QTL also influences body weight and the quantitative trait nucleotides (QTN) modulate the expression of the surrounding genes, including *PLAG1*[11], whose knockout in mice causes dwarfism in the absence of other symptoms [12]. The *CW-1* QTL that we narrowed down [2] locates approximately 2.5-Mb apart from the variations on *RPS20* or *PLAG1-CHCHD7*.

Here, a GWAS for carcass weight was performed using 1156 Japanese Black steers that were selected preferentially from the tails of the carcass weight distribution of more than 27,500 steers. The strongest association was obtained on BTA14, but not within the *CW-1* region. The GWAS led us to re-examine the *CW-1* QTL and identify the *PLAG1-CHCHD7* QTN for stature [11] in Japanese Black cattle.

## Results

### GWAS identified three major QTL in Japanese Black

**Figure 1:**
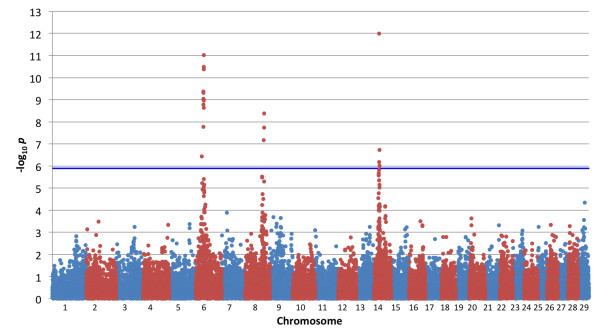
**Manhattan plot of GWAS for bovine carcass weight.** We genotyped 1156 Japanese Black steers with the Illumina Bovine SNP50 BeadChip. The association of SNP with carcass weight was analyzed by a variance component approach using EMMAX software [13] with adjustments for age, slaughterhouse, and year as covariates and fixed effects. Blue horizontal line, *p* = 1.28 × 10^-6^.

A GWAS was performed using 1156 DNA samples that were selected from more than 27,500 Japanese Black steers. The carcass weight of the selected samples had higher ratios of both extremes than in the collected samples, but was normally distributed (Additional file 1). The 39,011 SNP on autosomes that fulfilled the quality control criteria were used for the association analysis. The analysis was performed using EMMAX software [13], which is based on a linear mixed model approach using a genetic relationship matrix estimated by high-density SNP genotypes to model the correlation between the phenotypes of the sample subjects. The approach can adjust for both population stratification and relatedness between samples [14]. The genomic inflation factor (λ_GC_) was 1.008 in this analysis, indicating that the adjustment of the sample structure of our samples was appropriate. The quantile-quantile (Q-Q) plot with *p* < 10^-3^ revealed large deviations from the distribution under the null hypothesis, indicating that robust associations were obtained (Additional file 2). Bonferroni-corrected genome-wide significant associations (*p* < 1.28 × 10^-6^) were obtained for 19 SNP on 3 chromosomes: 12 on BTA 6, 3 on BTA 8, and 4 on BTA 14 (Figure 1; Additional file 3).

**Figure 2:**
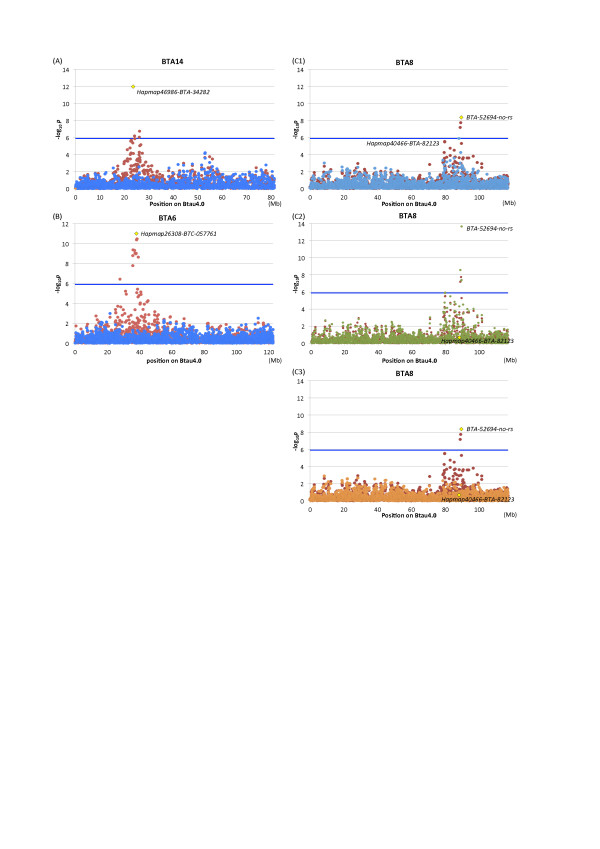
**Conditioned analyses.** To examine whether the associations detected on a chromosome were independent, a conditioned analysis was performed by including a genotype of the most significant SNP on the chromosome as a covariate in the model. Red and blue dots represent *p* values in –log_10_ scale before and after conditioning, respectively. Large squares indicate the most significant SNP in the respective chromosomes. (A) BTA 14. (B) BTA 6. (C1) BTA 8. In BTA 8, *Hapmap40466-BTA-82123* reached nearly genome-wide significance with conditioning on *BTA-52694-no-rs* (C1), while the association of *BTA-52694-no-r* became stronger with conditioning on *Hapmap40466-BTA-8212* (C2, green dots). When conditioned on both SNP, associations of the surrounding SNP disappeared (C3, orange dots).

The strongest association was detected with *Hapmap46986-BTA-34282* (*p* = 1.03 × 10^-12^) located at 23.5 Mb on BTA 14 (Btau4.0; Figure 2A; Table 1). This and three other significant SNP located from 23.5 to 26.2 Mb on BTA 14 were in moderate linkage disequilibrium (LD) with linkage disequilibrium coefficients (*r*^2^) ranging from 0.23 to 0.79. The second strongest association was obtained with *Hapmap26308-BTC-057761* (*p* = 9.65 × 10^-12^) on BTA 6, which had 11 other significant SNP in the interval (Figure 2B; Table 1). Eleven SNP, including *Hapmap26308-BTC-057761,* located within 3.5 Mb from 35.7 to 39.2 Mb and were in strong LD with *r*^2^ ranging from 0.65 to 1.00. A residual SNP was 7.9 Mb from them, but in strong LD with the 11 SNP (*r*^2^ = 0.62-0.73). An extended LD of this region was also observed in a previous study [3], which is probably due to a specific (local) lineage of the *Q* allele. On BTA 8, 3 SNP locating from 88.7 Mb to 89.4 Mb reached genome-wide significance (Figure 2C). The most significant SNP was *BTA-52694-no-rs* (*p* = 4.24 x 10^-9^; Table 1). In this case, the LD among the 3 SNP was relatively weak with *r*^2^ ranging from 0.14 to 0.35.

**Table 1:** Summary of the SNP and haplotypes associated with carcass weight

Category^1)^	SNP or Haplotype	Chr	Position (Btau4.0)	Allele substitution effect (kg)	*p-*value	*Q* allele	*q* allele	*Q* frequency
A	*Hapmap26308-BTC-057761*	6	37,963,147	35.5	9.65E-12	A	G	0.199
	*BTA-52694-no-rs*	8	89,397,242	26.0	4.24E-09	A	G	0.138
	*Hapmap46986-BTA-34282*	14	23,519,449	27.2	1.03E-12	A	G	0.759
B	*NCAPG* c.1326T > G	6	38,164,357	35.3	5.28E-12	G	T	0.200
	*Hapmap40466-BTA-82123* and *BTA-52694-no-rs*	8	-	35.0	8.13E-13	C-A	Others	0.115
	*FJX_PLAPROTRI*	14	23,264,810	28.4	2.84E-14	(CCG)_11_	(CCG)_9_	0.754

^1)^A, SNP on the BovineSNP50 BeadChip that showed the strongest association on a chromosome.

^1)^B, Critical SNP or a haplotype in the associated regions.

We then performed conditioned analyses to examine the independence of the SNP associations. Genotypes of the most associated SNP on each chromosome, *Hapmap26308-BTC-057761* on BTA 6, *BTA-52694-no-rs* on BTA 8, and *Hapmap46986-BTA-34282* on BTA 14, were individually included as a covariate in the mixed model. After conditioning, the associations of the surrounding SNP disappeared (Figure 2A, B), indicating that a single QTL was present in the respective regions. In the case of BTA 8, the *p* value of *Hapmap40466-BTA-82123* decreased from 0.216 to 1.31 × 10^-6^, a nearly genome-wide significance level, after conditioning on *BTA-52694-no-rs*, while *BTA-52694-no-rs* became much more significant (*p* = 2.17 × 10^-14^) after conditioning on *Hapmap40466-BTA-82123*. When conditioned on both *BTA-52694-no-rs* and *Hapmap40466-BTA-82123*, the associations of the surrounding SNP disappeared (Figure 2C). This result suggested that a certain haplotype comprising the two SNP represented the QTL on BTA 8 and that a SNP in strong LD with the causative variation of the QTL was not included in the BovineSNP50 BeadChip. When conditioned on the four SNP described above, the Q-Q plot did not deviate from the distribution under the null hypothesis, indicating that no other QTL were detected (Additional file 2).

In our half-sib analyses, we previously detected carcass weight QTL on BTA 14 (*CW-1*) [1,2,15,16], BTA 6 (*CW-2*) [1,3], and recently on BTA 8 (*CW-3*; Additional file 4). As for *CW-2*, the SNP encoding NCAPG Ile442Met has been identified as a candidate causative variation [3]. The SNP was located at 38.2 Mb and was in strong LD with *Hapmap26308-BTC-057761* (*r*^2^ = 0.97), indicating that the observed association corresponded to *CW-2* (Figure 2B; Table 1). On BTA 8, we detected a carcass weight QTL around 90 cM in two half-sib families (Additional file 4), whose sires are father and son and shared a superior *Q* haplotype (data not shown). Because the *Q* haplotype of the sires had an A-allele for *BTA-52694-no-rs* and a C-allele for *Hapmap40466-BTA-82123*, the haplotypes comprising the two SNP were estimated for the GWAS samples and the association of the *Q* haplotype was examined (Table 1). The *Q* haplotype was highly associated with carcass weight (*p* = 8.13 × 10^-13^), indicating that the association detected on BTA 8 corresponded to *CW-3*. In contrast, the 1.1-Mb critical region for *CW-1*[2] was 2.3-Mb telomeric from the most strongly associated SNP on BTA 14 in the GWAS. The details are discussed below.

### LD mapping led to a revision of a *CW-1* critical region

The *CW-1* QTL was originally mapped between *BMS1941* and *INRA094* (24.7-30.9 Mb on Btau4.0) using a Japanese Black half-sib family [15], and narrowed down between *DIK7012* and *DIK7020* (25.8-26.7 Mb on Btau4.0) by LD mapping [2]. A subsequent haplotype analysis showed that the six sires segregating a carcass weight QTL around the *CW-1* locus shared the same *Q* haplotype between *DIK7013* and *NRKM-040* (26.0-26.7 Mb on Btau4.0) with the *CW-1*-segregating sire [1], indicating that all seven sires harbored the *CW-1* QTL. The fact that many sires harbored the *CW-1* QTL suggested that the QTL was prevalant in the Japanese Black population. Despite this, the most significant SNP on BTA 14 in the GWAS (*Hapmap46986-BTA-3428* at 23.5 Mb on Btau4.0) was slightly outside of the confidence interval (CI) of *CW-1* and the conditioned analysis of the GWAS showed only one significant QTL on BTA 14. Thus, we performed haplotype analysis and LD mapping using high-density microsatellites developed around *Hapmap46986-BTA-34282*, to examine if the observed association was derived from *CW-1*, and if so, which location *CW-1* lies in. Haplotypes were examined using the three sires that segregated the *CW-1* QTL and harbored the same *Q* but different *q* (inferior) haplotypes between *DIK7013* and *NRKM-040*[1]. All three sires shared the same *Q* haplotype spanning more than 1.1 Mb around *Hapmap46986-BTA-34282* (Additional file 5). Therefore, the two identity-by-state (IBS) regions located 2.4-Mb apart on the *Q* haplotypes of the *CW-1*-segregating sires, which implied that the observed association was consistent with *CW-1*. The sires also shared the same *q* alleles at the two consecutive microsatellites close to *Hapmap46986-BTA-34282* (Additional file 5).

**Figure 3:**
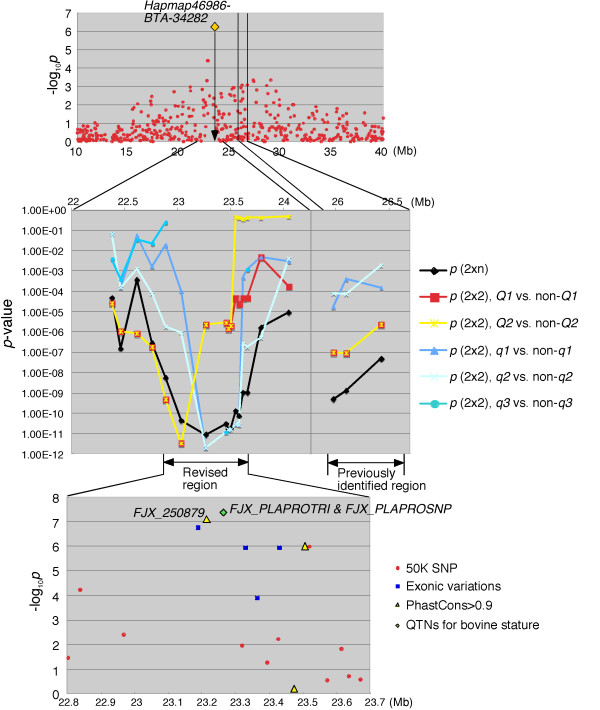
**Linkage disequilibrium mapping of*CW-1*and association analysis of the candidate QTN screened by targeted resequencing.** Of the 1156 steers used for GWAS, 142 with heavy carcass weight and 145 with light carcass weight were used. Also in this population, *Hapmap46986-BTA-34282* was the most associated on BTA 14 (*upper panel*). Linkage disequilibrium mapping was performed using high-density microsatellites around *Hapmap46986-BTA-34282* and the previously identified *CW-1* region. A haplotype frequency with two contiguous microsatellites was estimated using an expectation-maximization algorithm in each group and differences in haplotype frequencies between the groups were tested by Fisher’s exact test using a 2x*n* or a 2x2 contingency table (*middle panel*). The frequency of the *q3* haplotype was very low (< 6 alleles in both groups) in some regions, in which the association of the haplotype was not tested. Targeted resequencing was performed on the revised *CW-1* region. Candidate QTN were obtained as described in the text. An association of candidate QTN with carcass weight was examined using EMMAX software [13] (*lower panel*).

Linkage disequilibrium mapping was performed using 142 cattle with heavy carcass weight and 145 cattle with light carcass weight that were selected from the GWAS samples and did not include half-sibs in either group. Also, in the selected samples, *Hapmap46986-BTA-34282* had the strongest association on BTA 14 (Figure 3*upper*). The frequency of haplotypes comprising two contiguous microsatellites was estimated in each group and the difference in haplotype frequencies between the groups was tested by Fisher’s exact test using 2x*n* or 2x2 contingency tables (Figure 3*middle*). A stronger association was detected around *Hapmap46986-BTA-34282* than the previously identified *CW-1* region in both tests using 2xn and 2x2 contingency tables, respectively, indicating that *CW-1* did not locate within the previously identified region, but located between *DIK7104* and *DIK7113* (22.8-23.7 Mb on Btau4.0; 24.6-25.4 Mb on UMD3.0). The revised *CW-1* region included both *RPS20* and *PLAG1-CHCHD7*, which were previously identified as a locus for body size [9] and stature [11], respectively.

### *CW-1* is identical to the QTN for bovine stature

To identify the causative variation of *CW-1*, targeted resequencing was performed. The three sires segregating the *CW-1* QTL, three *Q*-homozygous sires, and a *q*-homozygous steer were subjected to sequence capture using a NimbleGen custom array followed by resequencing using Illumina GAIIx (Illumina, San Diego, CA). The *Q*-homozygous sires were determined as those with at least one *Q* haplotype within the revised *CW-1* region and homozygous *Q* alleles at *Hapmap46986-BTA-34282* (Additional file 5), and did not segregate a carcass weight QTL on BTA 14 in the half-sib analyses using more than 255 offspring per family. The respective GAIIx-reads (40-bp paired-ends) were aligned against both Btau4.0 and UMD3.1 as reference genome sequences, because UMD3.1 includes the largest number of gene annotations among the existing bovine genome assemblies, while information about sequence conservation across species is available on Btau4.0 [17]. The mean sequence depth of the targeted region was estimated to be 26 to 59 per animal. On UMD3.1, 711 to 1309 putative sequence variations were identified in the *CW-1*-heterozygous sires. The obtained putative sequence variations were filtered by: (1) heterozygous in the three sires segregating the *CW-1* QTL, (2) homozygous in the three *Q*- and the *q*-homozygous animals, and (3) opposite alleles between the *Q*- and the *q*-homozygous animals, resulting in 211 candidate QTN (197 SNP and 14 Indel) on UMD3.1. There were 4 exonic variations, including 1 non-synonymous SNP, while 1 synonymous SNP and 5 SNP in the intergenic regions were within highly conserved segments in mammals (PhastCons score >0.9 on Btau4.0 [17]). We checked the sequence coverage of the coding regions and the highly conserved regions and confirmed that no other candidate QTN was present in them except for the *PLAG1CHCHD7* intergenic region (see Methods). The same variations (1 SNP and 1 variable number of tandem repeat) as those identified by Karim et al. [11] were detected in the *PLAG1CHCHD7* intergenic region of the *CW-1* heterozygous sires, while the SNP in a putative polyadenylation signal of *RPS20*[9] was homozygous in one of the *CW-1* heterozygous sires (Sire J in Additional file 5). Thus, a total of 11 sequence variations were examined for an association with carcass weight (Figure 3*lower*; Additional file 6). The strongest association was obtained for the variations between *PLAG1* and *CHCHD7*, strongly suggesting that *CW-1* was identical to the QTL for bovine stature identified by Karim et al. [11].

We compared haplotypes around the QTN for bovine stature between the Japanese Black sires and the F1 (Holstein x Jersey) sires reported by Karim et al. [11]. The *Q* haplotype of the Japanese Black sires had a mosaic structure of the *Q* and *q* haplotypes of the F1 sires, and shared the same *Q* alleles only at the QTN for stature, *FJX_PLAPROTRI* and *FJX_PLAPROSNP* (Additional file 7). This observation strongly supports the idea that a crossbreed comparison is a useful way to pinpoint the causative variation for a QTL in bovine [3] as well as in canine [18].

### The three major QTL accounted for one-third of the total genetic variance

Allele frequencies and effect size of the three QTL were examined using *FJX_PLAPROTRI* (*CW-1*), *NCAPG* c.1326T > G (*CW-2*), and a haplotype comprising *Hapmap40466-BTA-82123* and *BTA-52694-no-rs* (*CW-3*) (Table 1). The *Q* allele frequency was high for *CW-1* (75.4%), but low for *CW-2* (20.0%) and *CW-3* (11.5%), while allele substitution effects of *CW-2* and *CW*-*3* (35.3 and 35.0 kg/allele) were larger than that of *CW-1* (28.4 kg/allele). Thus, *CW-2* and *CW*-*3* can be used to efficiently increase carcass weight in the Japanese Black population.

The fraction of phenotypic variance explained by the empirically estimated relatedness matrix is calculated in EMMAX as pseudoheritability: $h^{2}=\sigma_{a}^{2}/\left(\sigma_{a}^{2}+\sigma_{e}^{2}\right)$. The value was 0.636 and decreased to 0.423 after conditioning on the 3 QTL (*FJX_PLAPROTRI*, *NCAPG* c.1326T > G and a haplotype comprising *BTA-52694-no-rs* and *Hapmap40466-BTA-82123*), suggesting that the 3 QTL account for one-third [=(0.636-0.423)/0.636] of the total genetic variance of the GWAS samples.

## Discussion

We detected three major QTL for carcass weight in a GWAS using Japanese Black cattle. Although these QTL were previously detected using microsatellite-based mapping in half-sib families, the allele frequencies and allele substitution effects of the QTL could be compared in the GWAS. Importantly, these three QTL alone accounted for approximately one-third of the genetic variance. Such information will be useful for marker-assisted selection.

The GWAS using a large population unexpectedly led us to revise the location of *CW-1.* The QTL mapping [15] was originally performed when only a medium-density linkage map was available. The limited number of microsatellites used for the fine-mapping may have caused the incorrect CI. The CI was 10.7 cM, while marker intervals around the QTL region were approximately 8.3 cM [15]. Once the CI was incorrectly assigned, an IBS region existing in the CI showed the strongest association in the following LD mapping population, and was assigned as a critical region [2]. To avoid the error, the fine-mapping should have been performed using high-density markers that were evenly distributed along the chromosome, as Karim et al. did [11]. They used 56 microsatellites in the fine-mapping, whose marker-density was more than 3 times the density of ours [15].

Re-examination of *CW-1* strongly suggested that it was identical to the causative variations for bovine stature that were identified by Karim et al. [11]. In their study, the QTL accompanied an effect on weight but not on the ratio of live weight to height [11], suggesting that the primary effect of *CW-1* may be on height as observed in *CW-2*[8].

In both *CW-1*[11] and *CW-2*[3,8], the same QTL mutations were shared among the historically and geographically distant breeds. To our knowledge, there is no documentation of a crossing between a European breed and an ancestor of the Japanese Black sires used in these studies. In *CW-1*, the *Q* haplotype of Japanese Black (Additional file 7) resembled a Simmental haplotype [11]. A close relationship has not been detected, however, between Japanese Black and a European breed, even using more than 2000 genome-wide SNP [19]. A genome-wide analysis of haplotypes using much higher-density SNP might reveal shared genomic fragments between the Japanese Black and a European breed, and a cryptic genomic influence from a certain breed. Although the origins of the QTN are unknown, it is important that the causative variations are shared among breeds at some QTL. A crossbreed comparison will be able to pinpoint the causative variation if it is shared.

The *CW-1* orthologous region in humans has been identified as one of the loci associated with adult height [7,10]. The *CW-2* also corresponds to another height locus, *NCAPG-LCORL*[3,8]. Pryce et al. [20] suggested the consistency between the loci for bovine stature and the loci for human adult height. Therefore *CW-3* may correspond to another height locus as well. The *CW-3* region includes many height-associated genes such as *C9orf64ZCCHC6SPIN1, IPPK, PTPDC1, PTCH1/FANCC, ZNF462,* and *LPAR*[21]. Targeted resequencing of the *CW-3* region will be useful to screen the candidate causative variations, as shown in this study.

So far, 180 loci have been detected that are associated with human adult height but they account for only 10% of the phenotypic variance [21]. Because heritability is estimated to account for more than 80% of the height variance [22], a large fraction of heritability has not been detected. In contrast, only six loci account for more than 70% of the variance in stature across domestic dog breeds [23]. Many of the loci are among the highest F_ST_ regions in the dog genome, suggesting that the simple genetic architecture resulted from diversifying selection among breeds for body size [23]. Under artificial selection, a QTL with a large effect such as *CW-1CW2*, and *CW3*, may be easily fixed. Previous reports showed that the *Q* frequencies of the stature QTL (*CW-1*) and *NCAPG* c.1326T > G (*CW-2*) differed substantially in P_0_ animals between Holstein-Friesian and Jersey [11] and between Charolais and German Holstein [24], respectively. In contrast, Japanese Black has three QTL at different frequencies. In Japanese Black, mean carcass weight was recently improved, while a line with a relatively small body size remains locally. This may explain the wide distribution of carcass weight in this breed (Additional file 1; 300–700 kg) and the presence of the three QTL with large effects. The line is known to produce highly marbled beef, but the *Q* alleles of the three QTL were not significantly associated with marbling (*p* >0.01). Therefore, the small carcass weight of the line can be improved by selection (*CW-1*) or introgression of the *Q* alleles (*CW-2* and *CW-3*) without affecting marbling. This study revealed a genetic architecture underlying carcass weight variations in Japanese Black cattle and provides useful information for breeding.

## Conclusions

Genome-wide association analysis of Japanese Black cattle identified three major QTL for carcass weight. A detailed examination of the association on BTA 14 led to a revision of the *CW-1* region and strongly suggested that it was identical to the causative variations in the *PLAG1-CHCHD7* intergenic region for bovine stature [11]. The three QTL with various frequencies accounted for approximately one-third of the total heritability, indicating a possibility of efficiently increasing carcass weight using marker-assisted selection.

## Methods

### Ethics statement

This research was undertaken with the approval of the Shirakawa Institute of Animal Genetics Committee on Animal Research (H21-1).

### Collection of DNA samples and phenotype data

Perirenal fat tissues of more than 27,500 Japanese Black steers were collected at two slaughterhouses from 2000 to 2008. The cattle were reared in different herds throughout Japan. Carcass traits such as cold carcass weight and marbling were systematically measured by certified graders and recorded at slaughterhouses. Sire DNA used to analyze the *CW-1* region was obtained as semen.

### Selection of DNA samples for GWAS

First, animals were selected from the 15% extremes of the cold carcass weight distribution that did not include a half-sib in each extreme, resulting in 199 steers for heavy carcass weight and 299 steers for light carcass weight. Then, 658 steers that had been selected from both extremes for marbling and genotyped using the BovineSNP50 BeadChip (Illumina) were added to increase the sample numbers. The 658 steers included half-sibs and distributed from heavy to light carcass weight. The resulting population comprised 1156 steers with a normally distributed carcass weight (Additional file 1). The 1156 steers were the offspring of 347 sires and included at most 20 half-sibs.

### GWAS for carcass weight

The 1156 DNA samples were genotyped using the BovineSNP50 BeadChip (Illumina). The Btau4.0 assembly [25] was used to map the position of the SNP. Genotyping call rates were more than 95% in all samples. Among 54,001 SNP on the BovineSNP50 BeadChip, 39,011 SNP on autosomes fulfilled our quality control criteria: (1) call rate greater than 95%, (2) minor allele frequency greater than 0.01, and (3) p-value of a chi-square test for Hardy-Weinberg equilibrium greater than 0.001.

An association analysis was performed using EMMAX software [13] based on a linear mixed model with genomic related matrix. This software performs three-step analysis. First, the n × n genetic relatedness matrix of IBS between individuals is calculated using high-density SNP genotype data. Second, it uses a variance component model to estimate the restricted maximum likelihood parameters.

(1) $$Var\left(Y\right)=\sigma_{a}^{2}{\hat{S}}_{N}+\sigma_{e}^{2}I$$

where *Var*(*Y*) is the variance of phenotype, *σ*_*a*_^2^ is the additive genetic variance, ${\hat{S}}_{N}$ is the n × n normalized genetic relatedness matrix, *σ*_*e*_^2^ is random environmental variance, and *I* is an identity matrix.

Third, a generalized least squares *F*-test for each marker was performed to detect associations accounting for the sample structure using the genetic relatedness matrix

(2) $$y_{i}=\beta_{0}+\beta_{k}X_{\mathrm{ik}}+\eta_{i}\;Var\left(\eta \right)=V\propto \sigma_{\hat{a}}^{2}{\hat{S}}_{N}+\sigma_{\hat{e}}^{2}I$$

where *y*_*i*_ is the phenotypic value of an individual *iβ*_*0*_ is the fixed effect composed of age (month), slaughter year, and slaughterhouse to adjust the environmental effect, *β*_*k*_ is the effect size of marker *kX*_*ik*_ is the minor allele counts of marker *k* of individual *i*, and *η*_i_ is the error term that includes the genetic relatedness matrix. In a conditioned analysis, the genotypes of SNP associated with carcass weight were included as covariates. Haploview [26] was used to analyze LD among the SNP.

### QTL mapping of *CW-3*

The genome screen was conducted using the microsatellite markers on the Shirakawa-USDA linkage map [27]. QTL analyses were performed with the interval mapping method using a linear regression model for half-sib families [28,29], as described previously [15]. Linear regression analysis was performed using the following model:

(3) y=Xb+e,

where *y* is the vector of phenotypic value, *X* is the design matrix of fixed effects composed of slaughter year, age (day), and probability of having the *Q* phase at a given location (*Prob(Q)*), *b* is the vector of fixed effects, and *e* is the residual error. *b* was estimated by the least squares method. An *F*-statistic value at each position was calculated from the residual sum square regressed with *Prob(Q)*, and the total residual sum square without *Prob(Q)*. The analysis was performed at 2-cM intervals along each chromosome. A threshold for significance of the *F*-statistic value was obtained by 10,000 random permutations of the phenotypic data [30]. The 95% CI of the QTL locations was calculated using the bootstrapping method [31]. Briefly, a set of offspring was chosen so as to be the same number as the original half-sibs by resampling from the original half-sibs. Resampling was repeated 10,000 times. The position of the *F*-statistic peak in each bootstrapping was collected. The CI was determined based on the distribution of the peaks. Therefore, the CI may be fragmented into separated regions, and not a single contiguous region.

The sires and a *Q*-homozygous steer were genotyped using a BovineHD BeadChip (Illumina) that includes *BTA-52694-no-rs.* The SNP *Hapmap40466-BTA-82123* was genotyped by direct sequencing of the polymerase chain reaction (PCR) products using BigDye Terminator v.3.1 Cycle Sequencing Kit (Applied Biosystems) followed by electrophoresis using an ABI 3730 sequencer (Applied Biosystems).

### Microsatellite development and genotyping

Microsatellites were searched for in the genomic sequences and the primers were designed using Primer 3 [32]. Seventeen microsatellite markers from *DIK7100* to *DIK7116* were developed in this study. Marker information, such as primer sequences and genomic positions, is shown in Additional file 5. Genotyping was performed using PCR with a fluorescent-labeled reverse primer, followed by electrophoresis using ABI 3730 DNA analyzer (Applied Biosystems) and analysis using GeneMapper software (Applied Biosystems). The sires and their offspring were genotyped to determine the phase of the sires’ chromosomes. The haplotypes of the animals used for targeted resequencing are also shown in Additional file 5.

### Linkage disequilibrium mapping

Linkage disequilibrium mapping was performed using 142 heavy carcass weight cattle and 145 light carcass weight cattle that were selected from the GWAS samples and did not include half-sibs in each group. A haplotype frequency comprising two contiguous microsatellites was estimated using an expectation-maximization algorithm in each group and the difference in haplotype frequencies between the groups was tested by Fisher’s exact test using a 2x*n* contingency table. The association of each haplotype of the three sires segregating the *CW-1* QTL was also tested by Fisher’s exact test using a 2x2 contingency table. The frequency of *q3* haplotypeAdditional file ( 5) was very low (< 6 alleles in both groups) in some regions, where an association of the haplotype was not tested.

### Targeted resequencing

Three sires segregating the QTL, three *Q*-homozygous sires, and a *q*-homozygous steer (Additional file 5) were subjected to sequence capture (NimbleGen custom array) followed by resequencing (Illumina GAIIx, 40-bp paired-end run). The *Q*-homozygous sires were determined as those that harbored at least one *Q* haplotype and homozygous *Q* alleles at *Hapmap46986-BTA-34282* and did not segregate a carcass weight QTL on BTA 14 in the half-sib analyses using more than 255 offspring per family. The revised *CW-1* region including 22.8-23.7 Mb on Btau4.0 and 24.5-25.5 Mb on UMD3.0 was targeted. Alignment and variant detection were performed using Illumina pipeline (CASAVA 1.7). As a reference genome assembly, UMD3.1 was used to identify variants in coding regions, while Btau4.0 was used to search variants in conserved segments across mammalian species. PhastCons scores were downloaded from the University of California, Santa Cruz web site [17]. Obtained putative sequence variations were filtered by: (1) heterozygous in the three sires segregating the *CW-1* QTL, (2) homozygous in the three *Q*- and the *q*-homozygous animals, and (3) opposite alleles between the *Q*- and the *q*-homozygous animals.

Coverage of the coding regions (UMD3.1) and highly conserved regions (PhastCons > 0.9, Btau4.0) was checked in each animal. The region between *PLAG1* and *CHCHD7* was not covered in every animal. Although the region was difficult to amplify by PCR, we detected the variations in heterozygous sires reported by Karim et al. [11]. Ten nucleotides in the 3’UTR of *TMEM68* were not covered only in the *q*-homozygous animal and were sequenced using an ABI 3730 DNA analyzer (Applied Biosystems). Other coding regions and highly conserved regions were covered in every animal. Regions with fewer than 4 reads in either of the animals were confirmed to have no sequence variations between the *q*-homozygous and a *Q*-homozygous animal.

### Association analysis of candidate QTN with bovine carcass weight

Sequence variations were genotyped using the same population used for the LD mapping. The SNP were genotyped by direct sequencing of the PCR products, while the Indel and variable number of tandem repeat were amplified using a fluorescent-labeled reverse primer and genotyped in the same way as a microsatellite marker. The information, such as genomic positions, primer sequences, and alleles, is shown in Additional file 6. The genotypes were combined with the genotypes determined using a Bovine SNP50 BeadChip. Mixed-model association analysis was performed using EMMAX [13], adjusting for age, slaughterhouse, and year as covariate and fixed effects.

### Estimation of effect size of the QTL

Genotypes of *FJX_PLAPROTRI* and *NCAPG* c.1326T > G, and a *Q* haplotype comprising *Hapmap40466-BTA-82123* and *BTA-52694-no-rs* were used to estimate the effect size of *CW-1CW2,* and *CW3*, respectively. The 1156 GWAS samples were genotyped with *FJX_PLAPROTRI* (Additional file 6) and *NCAPG* c.1326T > G [3]. A *Q* haplotype of *CW-3* was determined using the sires segregating the QTL (Additional file 5) and diplotypes of the GWAS samples were estimated using fastPhase [33]. The calculation was performed using EMMAX including a number of the *Q* alleles or haplotypes (0, 1, and 2) as *X*_*ik*_ in the model.

## Abbreviations

QTL: Quantitative trait locus or loci; GWAS: Genome-wide association study; BTA: Bovine chromosome(s); SNP: Single nucleotide polymorphism(s); QTN: Quantitative trait nucleotide(s); LD: Linkage disequilibrium; Q-Q: Quantile-quantile; IBS: Identity-by-state; CI: Confidence interval; PCR: Polymerase chain reaction.

## Competing interests

The authors declare that they have no competing interests.

## Authors’ contributions

SN and TW performed the statistical analyses. KM and KT contributed in the reanalysis of the *CW-1* region. TF and NW mapped *CW-3* in half-sib QTL analyses. YS coordinated the study and helped draft the manuscript. AT designed the study and performed targeted resequencing. AT and SN drafted the manuscript. All authors read and approved the final manuscript.

## Supplementary Material

Additional file 1 Distribution of carcass weight of the animals used for the GWAS. This figure shows the distributions of carcass weight in the collected (blue bars) and the GWAS samples (red bars), respectively. Click here for file

Additional file 2 Quantile-quantile (Q-Q) plots of GWAS. The figures show Q-Q plots before (red) and after conditioned analyses (blue). Click here for file

Additional file 3 SNP associated with carcass weight. The table shows genome-wide significant SNP. Click here for file

Additional file 4 *F*-statistic profiles for carcass weight on BTA 8. This file shows *F*-statistic profiles for carcass weight on BTA 8 obtained in two QTL analyses. The QTL analyses were performed as described previously [1]. The two sires are father (Sire I) and son (Sire II), and the *Q* haplotype was inherited by the son from the father (data not shown). Marker locations were obtained from the Shirakawa-USDA linkage map [27]. Boxes on the x-axis indicate the 95% confidence interval of the QTL. Horizontal lines indicate the thresholds for chromosome-wise 0.1% (− − −), 1% (− −), and 5% (—–) significance levels. Click here for file

Additional file 5 Microsatellite markers and haplotypes of the animals used for targeted resequencing. This table shows marker information, such as primer sequences and physical positions, as well as haplotypes of the animals used for targeted resequencing. Click here for file

Additional file 6 Candidate QTN examined for an association with carcass weight. This table shows the information on sequence variations such as genomic positions, primer sequences, and *Q* and *q* alleles in the Japanese Black sires segregating *CW-1*. Click here for file

Additional file 7 Haplotypes of Japanese Black sires. This table shows *Q* and *q* alleles in Japanese Black sires around the causative variations for bovine stature. Click here for file
