# Supplementary figures and images for: Genome-wide association study identified three major QTL for carcass weight including the PLAG1-CHCHD7 QTN for stature in Japanese Black cattle
Source: BMC Genet. 2012 May 20;13:40. doi: 10.1186/1471-2156-13-40 (PMC3403917; doi:10.1186/1471-2156-13-40)

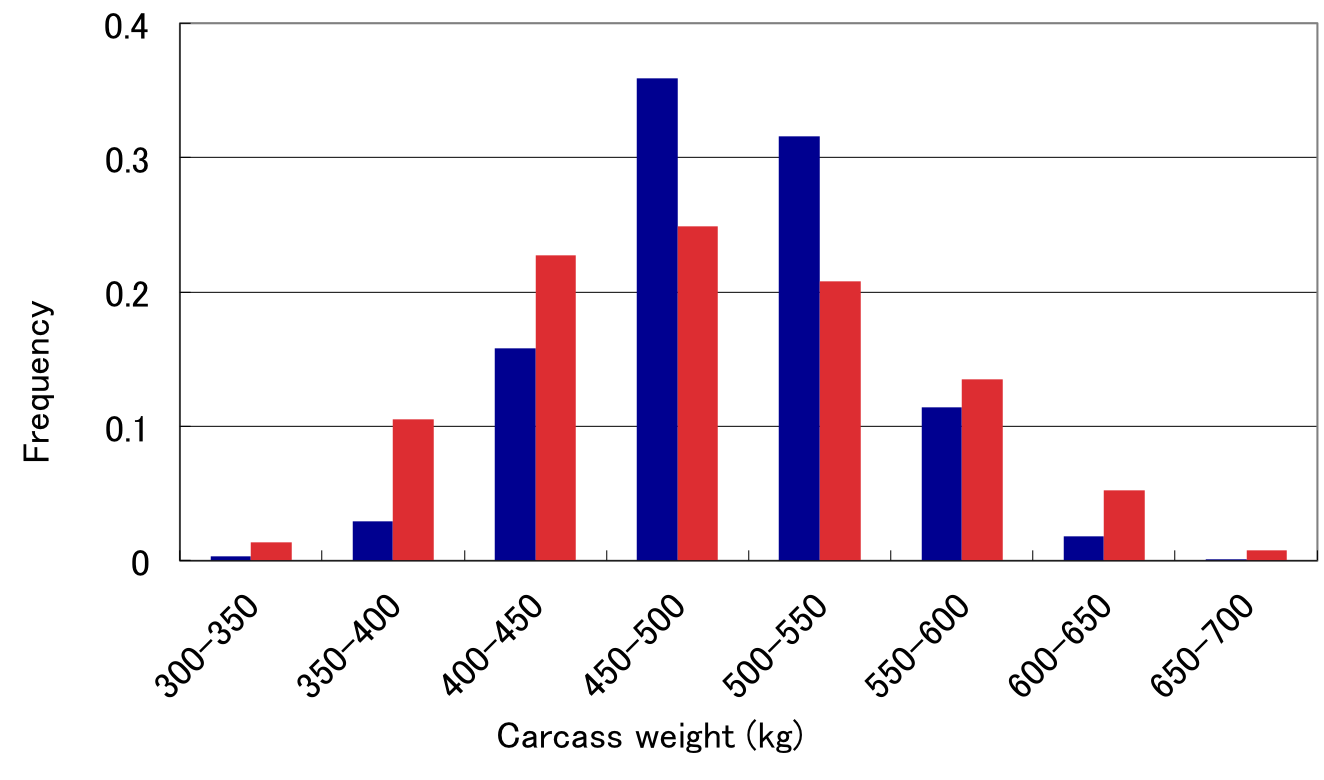

Supplement: Additional file 1 — Distribution of carcass weight of the animals used for the GWAS. This figure shows the distributions of carcass weight in the collected (blue bars) and the GWAS samples (red bars), respectively. [file 1471-2156-13-40-S1.png]

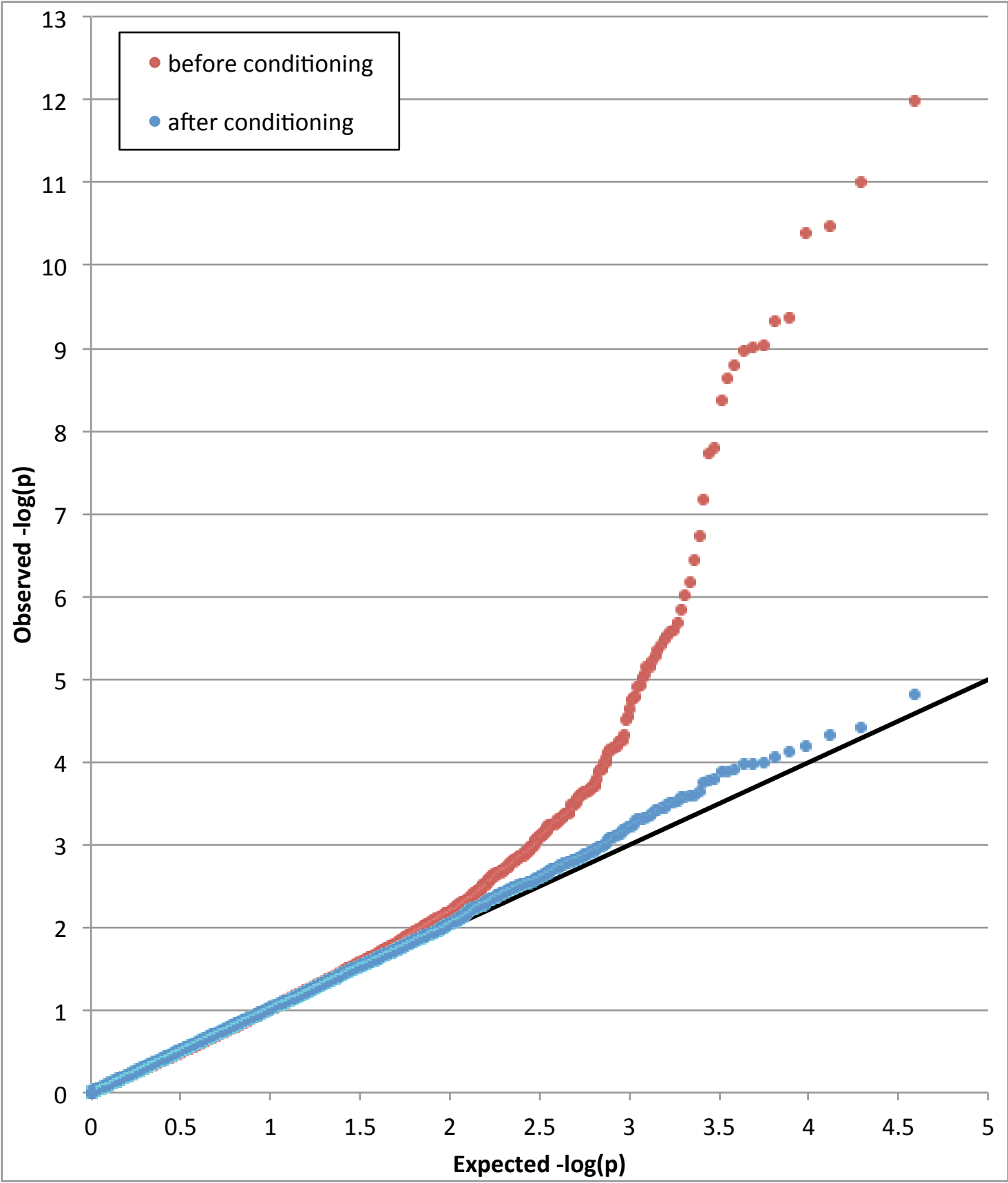

Supplement: Additional file 2 — Quantile-quantile (Q-Q) plots of GWAS. The figures show Q-Q plots before (red) and after conditioned analyses (blue). [file 1471-2156-13-40-S2.pdf]

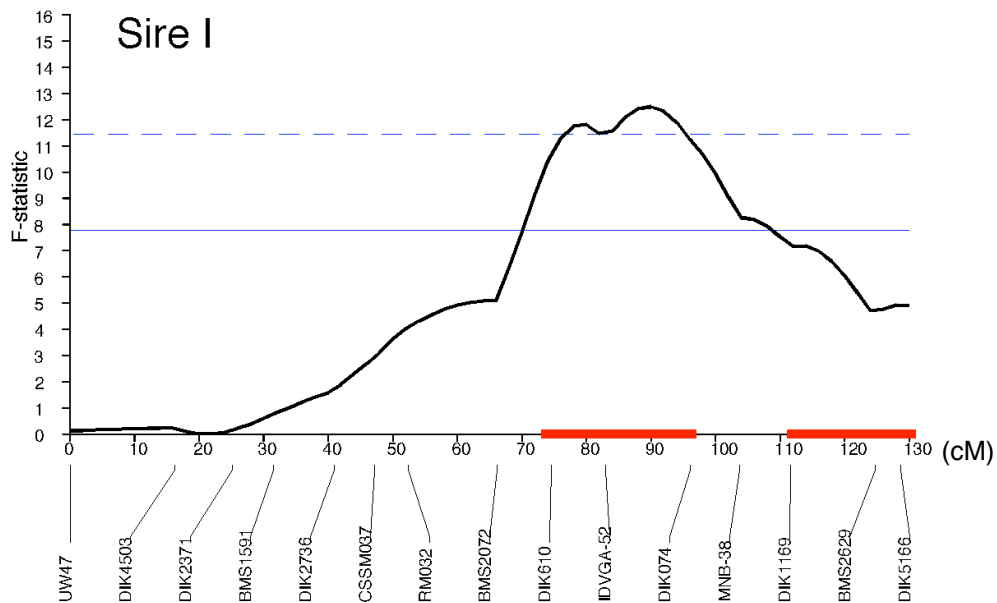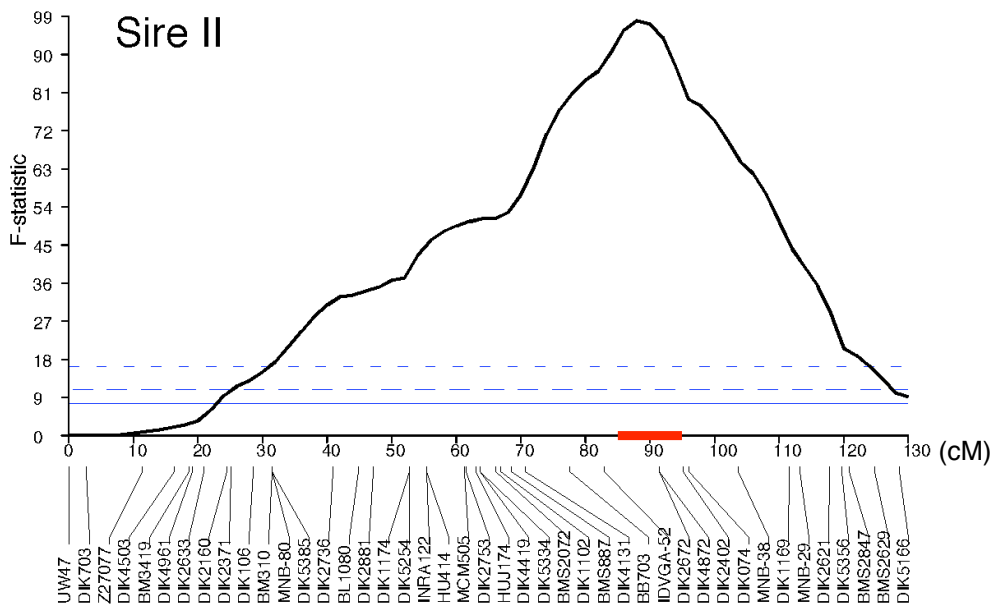

Supplement: Additional file 4 — F-statistic profiles for carcass weight on BTA 8. This file shows F-statistic profiles for carcass weight on BTA 8 obtained in two QTL analyses. The QTL analyses were performed as described previously [1]. The two sires are father (Sire I) and son (Sire II), and the Q haplotype was inherited by the son from the father (data not shown). Marker locations were obtained from the Shirakawa-USDA linkage map [27]. Boxes on the x-axis indicate the 95% confidence interval of the QTL. Horizontal lines indicate the thresholds for chromosome-wise 0.1% (− − −), 1% (− −), and 5% (—–) significance levels. [file 1471-2156-13-40-S4.pdf]
